# Azithromycin resistance in *Escherichia coli* and *Salmonella* from food-producing animals and meat in Europe

**DOI:** 10.1093/jac/dkae161

**Published:** 2024-05-22

**Authors:** Mirena Ivanova, Armen Ovsepian, Pimlapas Leekitcharoenphon, Anne Mette Seyfarth, Hanne Mordhorst, Saria Otani, Sandra Koeberl-Jelovcan, Mihail Milanov, Gordan Kompes, Maria Liapi, Tomáš Černý, Camilla Thougaard Vester, Agnès Perrin-Guyomard, Jens A Hammerl, Mirjam Grobbel, Eleni Valkanou, Szilárd Jánosi, Rosemarie Slowey, Patricia Alba, Virginia Carfora, Jelena Avsejenko, Asta Pereckiene, Dominique Claude, Renato Zerafa, Kees T Veldman, Cécile Boland, Cristina Garcia-Graells, Pierre Wattiau, Patrick Butaye, Magdalena Zając, Ana Amaro, Lurdes Clemente, Angela M Vaduva, Luminita-Maria Romascu, Nicoleta-Manuela Milita, Andrea Mojžišová, Irena Zdovc, Maria Jesús Zamora Escribano, Cristina De Frutos Escobar, Gudrun Overesch, Christopher Teale, Guy H Loneragan, Beatriz Guerra, Pierre Alexandre Beloeil, Amanda M V Brown, Rene S Hendriksen, Valeria Bortolaia, Jette Sejer Kjeldgaard

**Affiliations:** European Union Reference Laboratory for Antimicrobial Resistance (EURL-AR), Research Group for Global Capacity Building, Technical University of Denmark, Kongens Lyngby, Denmark; European Union Reference Laboratory for Antimicrobial Resistance (EURL-AR), Research Group for Global Capacity Building, Technical University of Denmark, Kongens Lyngby, Denmark; DIANA-Lab, Dept. of Computer Science and Biomedical Informatics, University of Thessaly, Lamia, Greece; Research Group for Genomic Epidemiology, Technical University of Denmark, Kongens Lyngby, Denmark; European Union Reference Laboratory for Antimicrobial Resistance (EURL-AR), Research Group for Global Capacity Building, Technical University of Denmark, Kongens Lyngby, Denmark; Research Group for Genomic Epidemiology, Technical University of Denmark, Kongens Lyngby, Denmark; Research Group for Genomic Epidemiology, Technical University of Denmark, Kongens Lyngby, Denmark; Austrian Agency for Health and Food Safety, Graz, Austria; National Diagnostic and Research Veterinary Institute, Sofia, Bulgaria; Croatian Veterinary Institute, Zagreb, Croatia; Bacteriology Serology Laboratory, Veterinary Services, Cyprus; State Veterinary Institute, Prague, Czech Republic; Danish Veterinary and Food Administration, Ringsted, Denmark; French Agency for Food, Environmental and Occupational Health & Safety, Maisons-Alfort, France; German Federal Institute for Risk Assessment, Berlin, Germany; German Federal Institute for Risk Assessment, Berlin, Germany; Veterinary Laboratory of Chalkis, Chalkis, Greece; National Food Chain Safety Office, Veterinary Diagnostic Directorate, Budapest, Hungary; Central Veterinary Research Laboratory, Kildare, Ireland; Istituto Zooprofilattico Sperimentale del Lazio e della Toscana ‘M. Aleandri’, Rome, Italy; Istituto Zooprofilattico Sperimentale del Lazio e della Toscana ‘M. Aleandri’, Rome, Italy; Institute of Food Safety, Animal Health and Environment BIOR, Riga, Latvia; National Food and Veterinary Risk Assessment Institute, Vilnius, Lithuania; Laboratoire de Médecine Vétérinaire de l’État, Dudelange, Luxembourg; Public Health Laboratory, Valletta, Malta; Wageningen Bioveterinary Research, Part of Wageningen University & Research, Lelystad, Netherlands; Sciensano, Brussels, Belgium; Sciensano, Brussels, Belgium; Sciensano, Brussels, Belgium; Department of Pathobiology, Ghent University, Merelbeke, Belgium; Jockey Club College of Veterinary Medicine and Life Sciences, Kowloon, Hong Kong; National Veterinary Research Institute, Pulawy, Poland; Instituto Nacional de Investigação Agrária e Veterinária, Oeiras, Portugal; Instituto Nacional de Investigação Agrária e Veterinária, Oeiras, Portugal; Institute for Hygiene and Veterinary Public Health, Bucharest, Romania; Institute for Diagnosis and Animal Health, Bucharest, Romania; Institute for Diagnosis and Animal Health, Bucharest, Romania; State Veterinary and Food Institute, Dolny Kubin, Slovakia; Institute for Microbiology and Parasitology, Ljubljana, Slovenia; Spanish Agency for Food Safety and Nutrition, Madrid, Spain; Spanish Agency for Food Safety and Nutrition, Madrid, Spain; Vetsuisse Faculty, Institute of Veterinary Bacteriology, University of Bern, Bern, Switzerland; Animal & Plant Health Agency, Weybridge, UK; School of Veterinary Medicine, Texas Tech University, Amarillo, TX, USA; European Food Safety Authority, Parma, Italy; European Food Safety Authority, Parma, Italy; Department of Biological Sciences, Texas Tech University, Lubbock, TX, USA; European Union Reference Laboratory for Antimicrobial Resistance (EURL-AR), Research Group for Global Capacity Building, Technical University of Denmark, Kongens Lyngby, Denmark; European Union Reference Laboratory for Antimicrobial Resistance (EURL-AR), Research Group for Global Capacity Building, Technical University of Denmark, Kongens Lyngby, Denmark; Statens Serum Institut, Copenhagen, Denmark; European Union Reference Laboratory for Antimicrobial Resistance (EURL-AR), Research Group for Global Capacity Building, Technical University of Denmark, Kongens Lyngby, Denmark

## Abstract

**Objectives:**

To characterize the genetic basis of azithromycin resistance in *Escherichia coli* and *Salmonella* collected within the EU harmonized antimicrobial resistance (AMR) surveillance programme in 2014–18 and the Danish AMR surveillance programme in 2016–19.

**Methods:**

WGS data of 1007 *E. coli* [165 azithromycin resistant (MIC > 16 mg/L)] and 269 *Salmonella* [29 azithromycin resistant (MIC > 16 mg/L)] were screened for acquired macrolide resistance genes and mutations in *rplDV*, 23S rRNA and *acrB* genes using ResFinder v4.0, AMRFinder Plus and custom scripts. Genotype–phenotype concordance was determined for all isolates. Transferability of *mef*(C)-*mph*(G)-carrying plasmids was assessed by conjugation experiments.

**Results:**

*mph*(A), *mph*(B), *mef*(B), *erm*(B) and *mef*(C)-*mph*(G) were detected in *E. coli* and *Salmonella*, whereas *erm*(C), *erm*(42), *ere*(A) and *mph*(E)-*msr*(E) were detected in *E. coli* only. The presence of macrolide resistance genes, alone or in combination, was concordant with the azithromycin-resistant phenotype in 69% of isolates. Distinct *mph*(A) operon structures were observed in azithromycin-susceptible (*n* = 50) and -resistant (*n* = 136) isolates. *mef*(C)-*mph*(G) were detected in porcine and bovine *E. coli* and in porcine *Salmonella enterica* serovar Derby and *Salmonella enterica* 1,4, [5],12:i:-, flanked downstream by IS*CR2* or Tn*As1* and associated with IncIγ and IncFII plasmids.

**Conclusions:**

Diverse azithromycin resistance genes were detected in *E. coli* and *Salmonella* from food-producing animals and meat in Europe. Azithromycin resistance genes *mef*(C)-*mph*(G) and *erm*(42) appear to be emerging primarily in porcine *E. coli* isolates. The identification of distinct *mph*(A) operon structures in susceptible and resistant isolates increases the predictive power of WGS-based methods for *in silico* detection of azithromycin resistance in Enterobacterales.

## Introduction

The macrolide azithromycin is a critically important clinical antimicrobial,^1^ increasingly used as an alternative when typical first-line antimicrobials (e.g. quinolones) are no longer effective in the treatment of severe cases of bacterial gastrointestinal infections.^2–5^ Considering that azithromycin is one of the few available options for treatment of MDR bacteria and that the majority of the azithromycin resistance genes are acquired, the spread of azithromycin resistance could seriously decrease the options to fight life-threatening infections. Among the azithromycin resistance genes,^6^  *mph*(A) and *erm* genes, encoding macrolide 2′-phosphotransferase and rRNA methylases, respectively, are the two main mechanisms involved in high-level azithromycin resistance.^7,8^ Another recently identified resistance mechanism in *Escherichia coli* conferred by tandemly arranged plasmid-borne genes *mef*(C)-*mph*(G), encoding an efflux pump and a phosphorylase, respectively, has been described to mediate high-level azithromycin resistance.^9^ Additionally, substitutions in the 50S ribosomal subunit proteins L4 (*rplD*) and L22 (*rplV*), in 23S rRNA (*rrlH*) and in the efflux pump AcrB (R717Q/L) also can lead to increased macrolide resistance.^2,6^ However, genes encoding efflux pumps, e.g. *msr*(A), *msr*(D), *mef*(A), *mef*(B), *ere* genes encoding macrolide esterases, and *mph*(B) encoding a macrolide phosphorylase appear to have no role or only marginal roles in azithromycin resistance in Enterobacterales.^6–8^

Due to the transmission of antimicrobial-resistant bacteria between animals and humans, the EU has implemented harmonized monitoring and reporting of antimicrobial resistance (AMR) in zoonotic and commensal bacteria from food-producing animals and food since 2014.^10,11^ In this monitoring programme, azithromycin susceptibility of *E. coli* and *Salmonella enterica* is tested phenotypically by broth microdilution.

Phenotypic azithromycin susceptibility testing is technically challenging and presents reproducibility issues in classifying isolates consistently as susceptible or resistant, which could be overcome by using WGS methods. However, these methods perform with high accuracy only for well-studied AMR determinants. Therefore, elucidating AMR gene patterns to increase the accuracy and effectiveness of WGS approaches is of great importance.

Although the prevalence of azithromycin-resistant *E. coli* and *Salmonella* in the EU has generally been reported to be low depending on the country and isolation source,^12^ a proper assessment of the risk to humans posed by azithromycin-resistant bacteria from food animals and food requires knowledge of resistance determinants. Therefore, the objectives of this study were to elucidate the genetic basis of azithromycin resistance in *E. coli* and *Salmonella* from food-producing animals and meat from 27 European countries and explore the azithromycin genotype–phenotype correspondence. Furthermore, this study aimed to resolve the molecular basis of genotype–phenotype discordance previously observed for *mph*(A), which is the most common azithromycin resistance gene in *E. coli*, and to characterize the genetic context and transferability of *mef*(C)-*mph*(G) genes, which are emerging in Enterobacterales.

## Methods

### Bacterial isolates

A total of 1276 isolates were examined in this study. These isolates comprised 1007 *E. coli* and 269 *Salmonella enterica* subsp. *enterica* serovars (Table S1, available as Supplementary data at *JAC* Online) collected within the EU harmonized monitoring of AMR in zoonotic and indicator bacteria from food-producing animals and food in 2014–18,^10,13^ and the Danish Programme for surveillance of antimicrobial consumption and resistance in bacteria from food animals and food (www.danmap.org) in 2017–19. The EU harmonized monitoring system involved biannual surveillance of different animal types. Isolates from 2014, 2016 and 2018 originated from poultry caeca and meat, whereas isolates from 2015 and 2017 and from the Danish AMR surveillance programme were recovered from porcine and bovine caeca and meat. For the Danish collection, only isolates with azithromycin MIC > 16 mg/L were included. Antimicrobial susceptibility testing was performed by broth microdilution using Sensititre MIC susceptibility plates (EUVSEC1 and EUVSEC2, Thermo Fisher Scientific) in duplicate, by the originating laboratory and at the EU Reference Laboratory – Antimicrobial Resistance (EURL-AR). In cases of more than one 2-fold dilution difference, the tests were repeated a third time. The azithromycin MIC values were interpreted in accordance with The European Food Safety Authority (EFSA)-defined surveillance breakpoint. Isolates with MIC ≤ 16 mg/L were classified as WT and isolates with MIC > 16 mg/L as non-WT.^13^

### Identification of genes and chromosomal mutations associated with macrolide resistance

WGS of the isolates was performed using Illumina paired-end sequencing on MiSeq, HiSeq or NextSeq platforms (Illumina, Inc., San Diego, CA, USA). Sequencing reads were trimmed using Trimmomatic v0.38^14^ and assembled by metaSPAdes v3.13.0.^15^ Assemblies were evaluated by Quast^16^ and genomes with ≤500 contigs were included in further analysis (Table S1). The ResFinder database v4.0^17^ incorporated into ABRicate v0.9.8 (https://github.com/tseemann/abricate) was used to detect acquired macrolide resistance genes with minimum identity and coverage thresholds of 90% and 60%, respectively. Chromosomal mutations in *acrB* and 23S rRNA genes were screened for using AMRFinder (https://github.com/ncbi/amr/wiki). The sequences of chromosomal *rplDV* genes were screened for mutations mediating azithromycin resistance^7,18^ using custom scripts. Sequence alignments of *rplDV* genes were performed using MAFFT in Geneious Prime v2020.0.4 (https://www.geneious.com) and compared with *rplDV* of *E. coli* ATCC 25922 (NC_000913.3) and *Salmonella enterica* serovar Typhimurium LT2 (NC_003197) as reference genes. MLST was performed using mlst v2.19.0 according to the Achtman schemes (https://github.com/tseemann/mlst). *Salmonella* serovar determination was carried out by SeqSero2 v1.1.1.^19^ Sequences from isolates positive for *mph*(A) and *mef*(C)-*mph*(G) were screened for plasmid replicon genes using PlasmidFinder (database version February 2020)^20^ with minimum identity and coverage thresholds as above. IS and Tn were identified using ISfinder (https://www-is.biotoul.fr)^21^ and TnCentral (https://tncentral.ncc.unesp.br/index.html). Visualization of the genetic contexts of the macrolide resistance genes was performed by pyGenomeViz v0.4.3 (https://github.com/moshi4/pyGenomeViz). Graphs were created using ‘ggplot2’ and ‘networkD3’ packages in R v4.1.0.^22,23^ Raw sequence data were submitted to the European Nucleotide Archive (ENA) at EMBL-EBI under accession numbers PRJEB18618, PRJEB21546, PRJEB33169, PRJEB43436, PRJEB43584, PRJEB63535 and PRJEB63683 (Table S1).

### Conjugation experiments and characterization of mef(C)-mph(G)-harbouring contigs

Isolates harbouring *mef*(C)-*mph*(G) (Table 1) were used as donors in filter-mating experiments with a rifampicin-resistant, lactose-negative *E. coli* J62-2 strain as recipient.^24^ Transconjugants (TCs) were selected on MacConkey agar (Sigma, Denmark) supplemented with 16 mg/L azithromycin (Sigma, Denmark) and 50 mg/L rifampicin (Sigma, Denmark). TCs were initially verified by MIC determination as described for the original isolates and by colony PCR targeting *mef*(C)-*mph*(G) using primers F3220-5′-ATTGGCGGTGTCATCCTGAG-3′ and R3221-5′-CGCTGACTTGTGCAGTTGAC-3′. Plasmid DNA from TCs with azithromycin MIC > 16 mg/L and *mef*(C)-*mph*(G) positive by PCR was extracted using the QIAGEN Plasmid Midi kit (QIAGEN, Germany), prepared for sequencing using the Nextera XT DNA Library Preparation Kit and sequenced with a NextSeq 500/550 Mid Output v2.5 Kit (300 cycles) on a NextSeq 500 platform (Illumina). Raw plasmid sequence data were subjected to quality checking using FastQC v0.11.5,^25^ trimmed with BBTools v.36.49^26^ and assembled by SPAdes v3.15.3.^27^ Assemblies were analysed by ResFinder and PlasmidFinder with thresholds as described above. Due to the common occurrence of co-extraction of chromosomal DNA during plasmid DNA extraction, seven-gene MLST analysis was successfully performed to distinguish between true TCs (having ST of the recipient strain) and mutated donor strains (having ST of the respective donor strain).

**Table 1.** Characteristics of *E. coli* and *Salmonella* sp. isolates carrying *mef(C)-mph(G)* genes

Strain ID	Species	ENA run accession number	Host	Country of origin	Source of isolation	Year of isolation	MLST ST	Azithromycin MIC (mg/L)	Conjugative transfer of *mef*(C)-*mph*(G)	Plasmid replicons^a^	Other AMR genes^a^
NRS-2015 ESBL-08-69	*E. coli*	ERR2019198	Porcine	Netherlands	Caecal content	2015	453	32	Yes	IncIFIB, IncX1, **IncIγ**, IncFII, IncQ1, **Col8282**	***tet*(A**), *sul3*, ***sul2***, *ant*(3′)-*Ia*, *cmlA1*, *aph*(3′)-*Ib*, *aph*(6)-*Id*, *dfrA5*, ***bla*_TEM-52_**
ZTA15:00420EB1	*E. coli*	ERR2019249	Porcine	Spain	Caecal content	2015	7456	32	No	ColE10, Col156, IncFII(pRSB107), Col(MG828), IncIγ, IncX4, IncHI2; IncHI2A, IncQ1	*tet*(M), *mcr-4.1*, *aph*(4)-*Ia*, *aac*(3)-*Ia*, *tet*(A), *bla*_CTX-M-14_, *bla*_TEM-1B_, *aph*(3′)-*Ia*, *mcr-1.1*, *mph*(B), *sul1*, *ant*(3′)-*Ia*, *drfA1*, *aph*(6)-*Id*, *aph*(3′)-*Ib*, *sul2*
HP-6957	*E. coli*	ERR3393110	Porcine	Czechia	Caecal content	2017	57	64	Yes	IncFIB, IncFIC(FII), **IncIγ**	*aadA1*
ECO NRS 187.57	*E. coli*	ERR3393279	Porcine	Netherlands	Caecal content	2017	88	>64	Yes	IncFIB, IncFIC(FII), **IncIγ**	—
17-AB00518_0	*E. coli*	ERR3393142	Bovine	Germany	Caecal content	2017	48	64	Yes	**IncIγ**, Col(pHAD28)	***sul2***, ***bla*_CTX-M-1_**
10047105/3	*E. coli*	ERR3393194	Porcine	Hungary	Meat	2017	453	>64	Yes	**IncFII**, IncIγ, IncY	***sul2***, *bla*_CTX-M-1_, ***tet*(A)**, *dfrA17*, *aadA5*
HP36996	*E. coli*	ERR3393109	Porcine	Czechia	Caecal content	2017	165	>64	No	IncFII, IncIγ, Col(MG828)	—
17-AB01235_0	*E. coli*	ERR3393151	Bovine	Germany	Caecal content	2017	NT^b^	32	No	IncFIA, IncFIB, IncFIC, IncIγ, IncX4	*sul2*, *tet*(B), *bla*_CTX-M-1_, *fosA7*
17-AB02375_0	*E. coli*	ERR3393160	Porcine	Germany	Caecal content	2017	57	64	No	IncFIA(HI1), IncFIB, IncFIC(FII), IncIγ, IncHI1A, IncHI1B	*sul2*, *sul3*, *bla*_CTX-M-1_, *tet*(A)*, tet*(B)*, dfrA12, dfrA1, qnrS1, aadA1, aadA2, aadA5, cmlA, floR, aph*(3′)-*Ib, aph*(3′)-*Ia, aph*(6)-*Id, bla*_TEM-1B_*, ant*(3′)-*1, tet*(M)
10849-2GR	*E. coli*	ERR3393337	Porcine	Romania	Caecal content	2017	101	64	No	Col156, ColRNAI, IncFIB, IncFIC(FII), IncIγ, IncFII, Col(pHAD28)	*sul2*, *bla*_CTX-M-1_, *tet*(A), *aadA5*, *aadA1, dfrA17, dfrA1, ant*(3′)-*Ia*
Ec151	*E. coli*	ERR11629170	Bovine	Denmark	Caecal content	2019	8863	>64	ND	IncIα	*sul2, aadA5, dfrA17, bla*_CTX-M-1_
S308	*Salmonella* 1,4,[5],12:i:-^c^	ERR11628334	Porcine	Denmark	Clinical	2017	19	>64	No	IncFIB, IncFII	*sul1*, *sul2*, *bla*_TEM-1B_, *aadA1*, *aac*(6′)-*Ia*; *tet*(A), *dfrA1*
S514	*Salmonella* Derby^d^	ERR11628347	Porcine	Denmark	Clinical	2018	40	>16	No	ColpVC, IncQ1	*sul2*, *aac*(6′)*-Ia; aph*(6)-*Id*, *aph*(3′)*-Ib*, *fosA7*, *dfrA17*

^a^In bold, plasmid replicons and AMR genes detected in TCs.

^b^Not typeable.

^c^ *S. enterica* subsp. *enterica* serovar 1,4, [5],12:i:-.

^d^ *S. enterica* subsp. *enterica* serovar Derby.

## Results and discussion

### Macrolide resistance genes in E. coli and Salmonella from food-producing animals and meat in the EU

**Figure 1.**
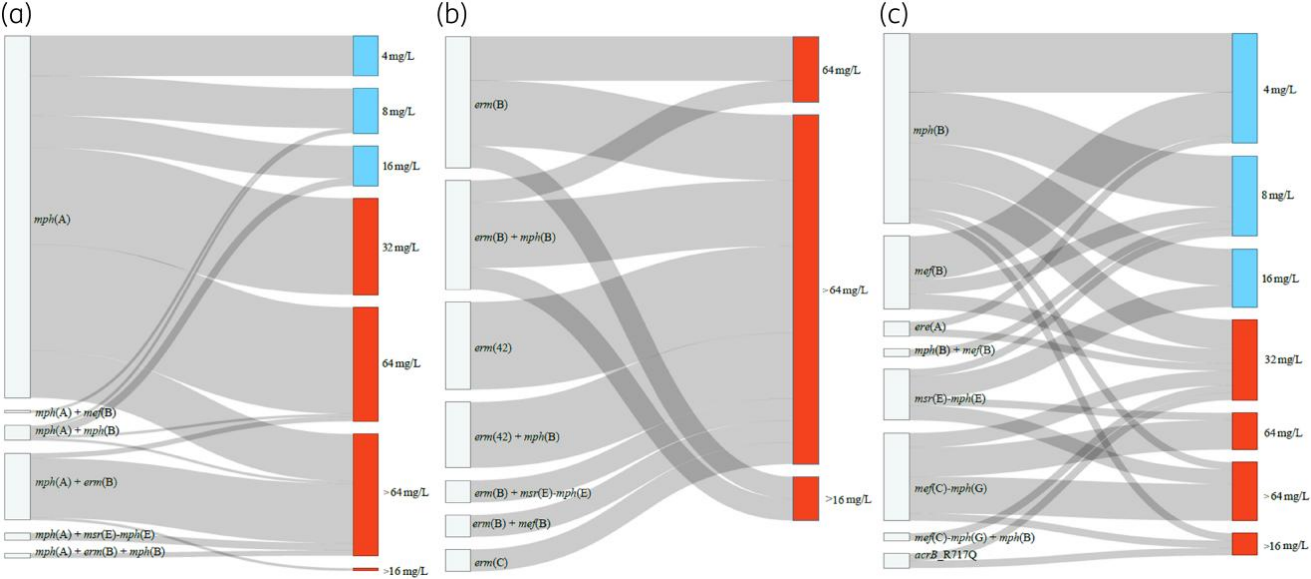
Sankey plots showing macrolide resistance genes/mutations and azithromycin MICs in the *E. coli* and *Salmonella* isolates in this study. *mph*(A) gene and its combinations with other macrolide resistance genes (a), *erm* genes and their combinations with other macrolide resistance genes (b), other macrolide resistance genes (c). The *rplDV* non-synonymous mutations detected in seven susceptible isolates were not included in the graph (Table S4). It is important to note that in resistant isolates harbouring *mph*(A) in combination with *mph*(B) or *mef*(B), the full *mph*(A) operon was present, and the promoter region was complete in all cases (Table S4). The 10 resistant isolates (MIC > 16 mg/L) without known azithromycin resistance mechanisms are not included in the graph. This figure appears in colour in the online version of *JAC* and in black and white in the print version of *JAC*.

**Figure 2.**
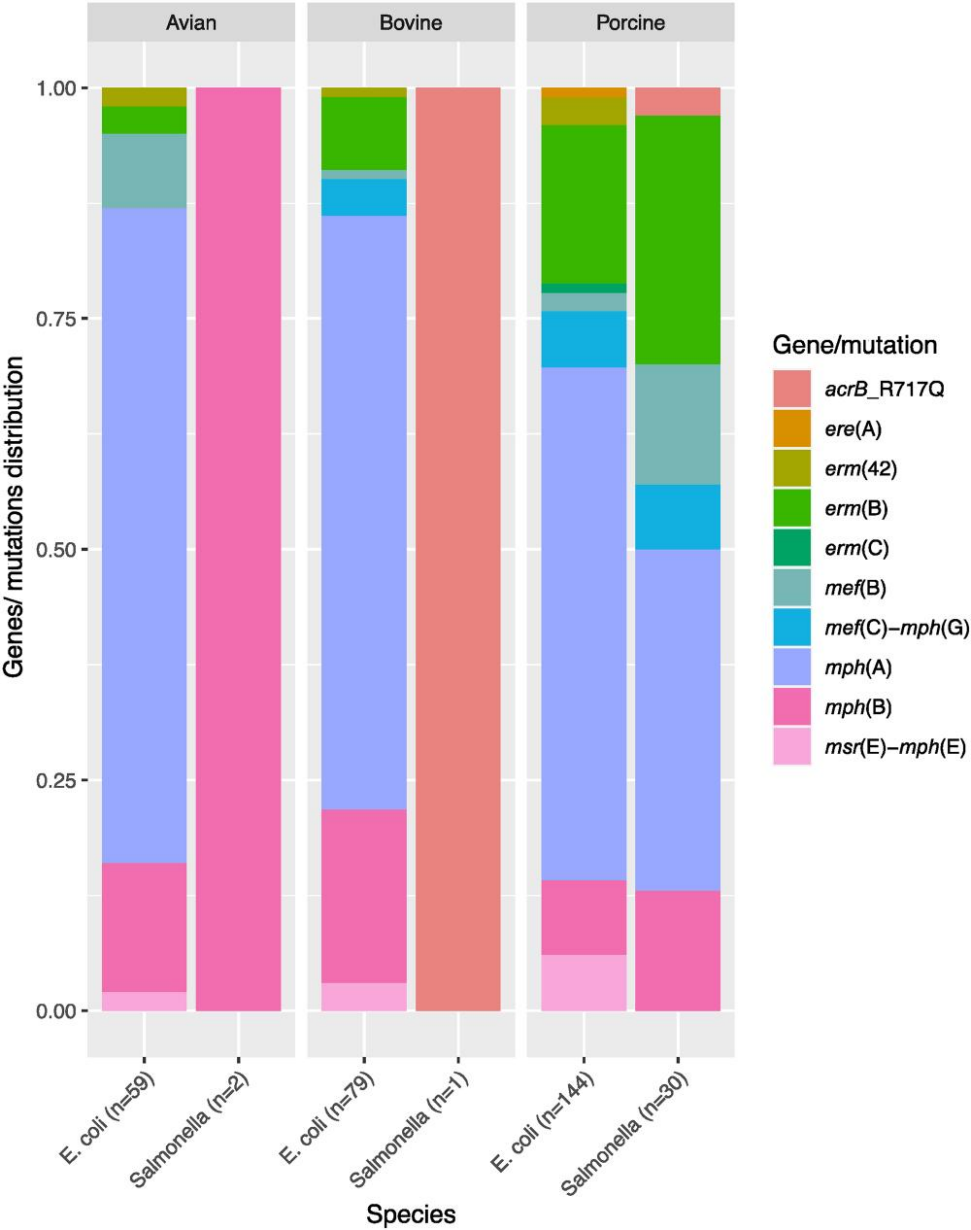
Distribution of macrolide resistance genes in *E. coli* and *Salmonella* isolates in this study according to their origin. Twenty-one percent (*n* = 51) of the isolates carried more than one macrolide resistance gene and are included under each of the genes they carry. The total number of genes in each species or from each source is given in parentheses. Two *mph*(A)- and two *mph*(B)-harbouring *E. coli* isolates for which isolation sources are not available are not included. This figure appears in colour in the online version of *JAC* and in black and white in the print version of *JAC*.

We screened commensal *E. coli* and *Salmonella* isolates from food-producing animals and meat in Europe for the presence of macrolide resistance determinants and compared these genotypes with their respective azithromycin phenotypes. Eleven different macrolide resistance genes and five non-synonymous *rplDV* mutations were detected in 248 *E. coli* and 26 *Salmonella* (*enterica* serovars *S*. Rissen, *S*. Blockley, *S*. Typhimurium, *S*. Paratyphi-B-var-Java, *S*. Bredeney, *S*. Infantis, *S.* 1,4, [5],12:i:-, *S*. Derby and *S.* Dublin) isolates (Tables S4, S5 and S6). The presence of one or more macrolide resistance gene(s) or known mutations was associated with the azithromycin resistance phenotype in 159 (66%) *E. coli* and 24 (92%) *Salmonella* isolates (Figure 1 and Table S4). No known genes or mutations mediating azithromycin resistance were detected in the remaining 1002 isolates, although 10 of these isolates exhibited azithromycin MIC > 16 mg/L (Table S4). Six genes, namely *mph*(A), *mph*(B), *mef*(B), *erm*(B) and *mef*(C)-*mph*(G), were detected in both *E. coli* and *Salmonella*, whereas *erm*(C), *erm*(42), *ere*(A) and *msr*(E)-*mph*(E) were detected in *E. coli* only (Table S4). Seven of all detected macrolide resistance genes [*mph*(A), *mph*(B), *mef*(B), *erm*(B), *erm*(42), *msr*(E)-*mph*(E)] were found in isolates from all animal sources, while *mef*(C)-*mph*(G) were detected in isolates of bovine and porcine origin only, and *ere*(A) and *erm*(C) were harboured by isolates of porcine origin only (Figure 2, Table S4).

Regarding chromosomal mutations, five non-synonymous *rplDV* mutations were detected in seven *E. coli* isolates with MIC ≤ 16 mg/L (Table S4), while no *rplDV* mutations were detected in *Salmonella*. Mutations in 23S rRNA genes were not detected in any of the analysed genomes. Two azithromycin-resistant *Salmonella* isolates (serovars 1,4, [5],12:i:- and Dublin) with no azithromycin resistance gene harboured the AcrB-R717Q substitution. The 10 isolates with no identified mechanism of azithromycin resistance were screened for additional non-synonymous mutations in the *acrR* and *acrB* genes, and either no mutations were found, or the detected amino acid substitutions/deletions were also observed in susceptible isolates (Table S4).

### Mph(A) operon structures in azithromycin-resistant and -susceptible isolates

One of the established macrolide resistance genes that confers high-level azithromycin resistance in Enterobacterales is *mph*(A).^5,6,28^ Here, *mph*(A) was detected in 15% of the isolates (*n* = 186). The *E. coli mph*(A)-harbouring isolates (*n* = 175) were genetically diverse, belonging to 77 STs, with ST744, ST1011, ST10 and ST410 being the most represented. The *mph*(A)-carrying *Salmonella* isolates (*n* = 11) belonged to four STs (ST469, ST34, ST19 and ST52) (Table S4). The isolates harbouring *mph*(A) displayed a wide range of azithromycin MIC values ranging from 4 to >64 mg/L (Figure 2 and Table S4). Of these isolates, 27% (*n* = 50) had a susceptible phenotype (MIC ≤ 16 mg/L). Previous studies have also shown that *mph*(A) can be present in azithromycin-susceptible isolates. For instance, Gomes *et al*.^7^ reported that 7% of the *mph*(A)-harbouring *E. coli* isolates in their study were susceptible, which suggests that the *mph*(A) gene does not always confer a resistant phenotype in Enterobacterales.

**Figure 3.**
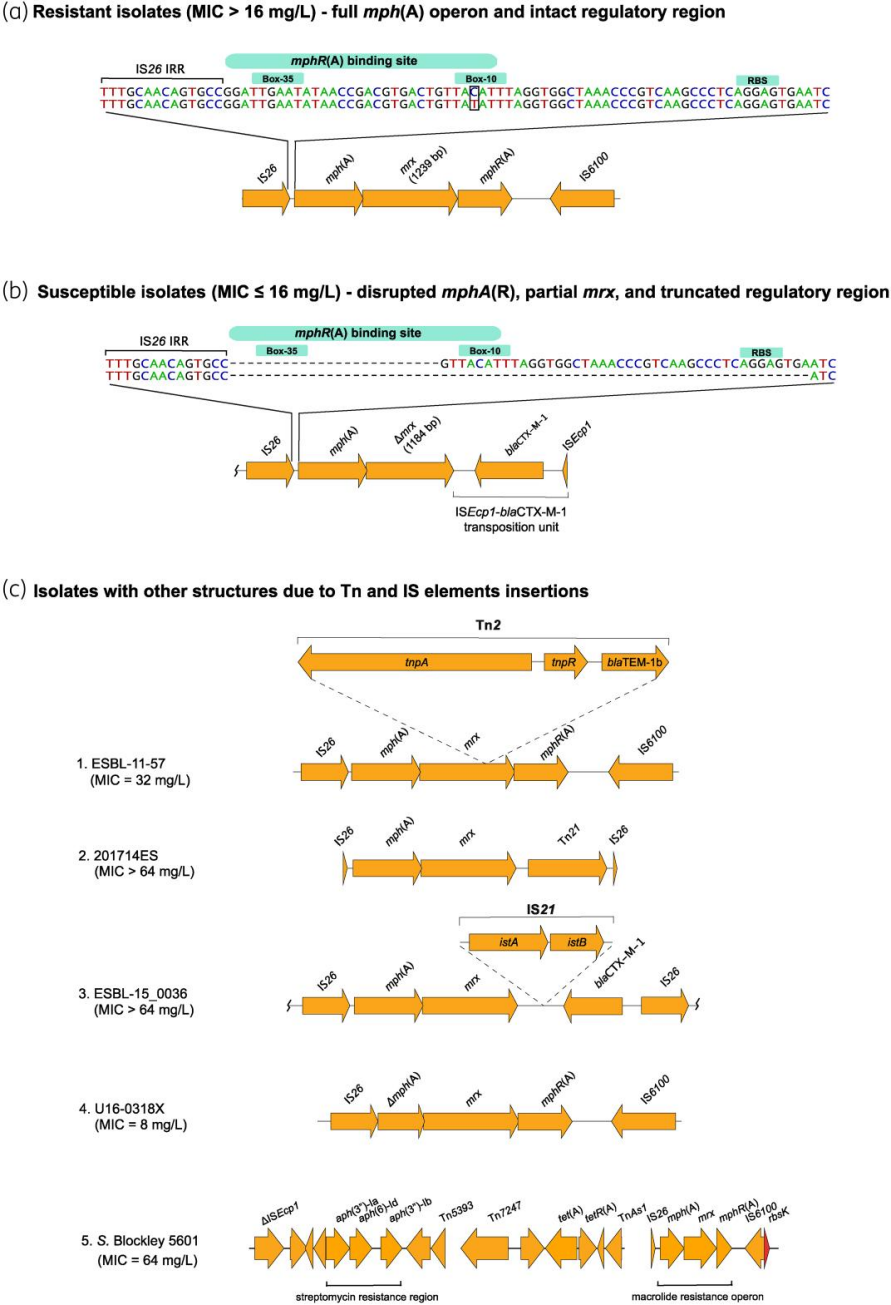
*mph*(A) operon structure in azithromycin-resistant isolates (MIC > 16 mg/L) (a), azithromycin-susceptible isolates (MIC ≤ 16 mg/L) (b), and in isolates with Tn and IS insertions (c). *S.* Blockley 5601 (c5) carries the *mph*(A) operon as part of an MDR region, consisting of the streptomycin resistance cluster *aph*(3′)-*Ib*–*aph*(6)-*Id*–*aph*(3′)-*Ia* and the *tet*(A) gene. NCBI blastn revealed 99.24%–100% identity and 100% coverage between the whole streptomycin and azithromycin resistance genomic region of *S.* Blockley isolate 5601 and *S.* Blockley strain 159383 (GenBank accession number CP043662.1: 4326383-4352831). The MDR region was reconstructed using Bandage (http://github.com/rrwick/Bandage). *istA*—IS*21*-like element IS*21* family transposase IstA, *istB*—IS*21*-like element IS*21* family helper ATPase IstB. In (a), the difference between the two sequences is T instead of C in the −10 region. Wavy lines at the end(s) of diagrams show where only part of the contig is included. This figure appears in colour in the online version of *JAC* and in black and white in the print version of *JAC*.

The *mph*(A) gene encoding macrolide 2′-phosphotransferase I is part of an operon, *mph*(A)-*mrx*-*mphR*(A), in which the downstream genes *mrx* and *mphR*(A) encode a protein with unknown function and a repressor that controls the inducible expression of *mph*(A), respectively.^29^ In this study, annotation of the complete *mph*(A) operon and analysis of the *mph*(A) promoter region, which overlaps with the *mphR*(A) binding site, revealed differences between azithromycin-susceptible and -resistant isolates. In general, resistant isolates (*n* = 136; 73%) had an intact *mph*(A) operon (Figure 3a), while *mph*(A)-harbouring susceptible isolates (*n* = 50; 27%) had altered operon structure (Figure 3b). Most commonly, susceptible isolates lacked the *mphR*(A) repressor gene and part of the *mrx* gene, disrupted by an IS*Ecp1*-*bla*_CTX-M-1_ transposition unit. Additionally, the susceptible isolates (*n* = 50) had either a 24 bp (*n* = 23) or a 66 bp (*n* = 14) deletion spanning the *mphR*(A) binding site and/or the transcription start site and the ribosomal binding site (RBS). Because it has been previously suggested that bacterial cells sense the presence of macrolides through the MphR(A) repressor by forming a macrolide–repressor complex,^29^ our interpretation of these results is that *mph*(A) is not expressed in susceptible isolates due to the lack of the macrolide–repressor complex.

Among the resistant *mph*(A)-harbouring isolates (*n* = 136), 117 followed the common *mph*(A) structure for resistant isolates [intact regulatory region and full *mph*(A) operon] (Figure 3a). One isolate (16037780A201X5, MIC = 32 mg/L) had a truncated regulatory region (Table S4) and three isolates had disruptions into the *mph*(A) operon due to Tn insertions (Figure 3c, 1–3). Additionally, four isolates had too short contigs to assess the completeness of the regulatory regions (Table S4). In *S.* Blockley 5601, the complete *mph*(A) operon was chromosomally integrated (Figure 3c, 5) as previously reported for *S.* Blockley strain 159383.^30^

**Figure 4.**
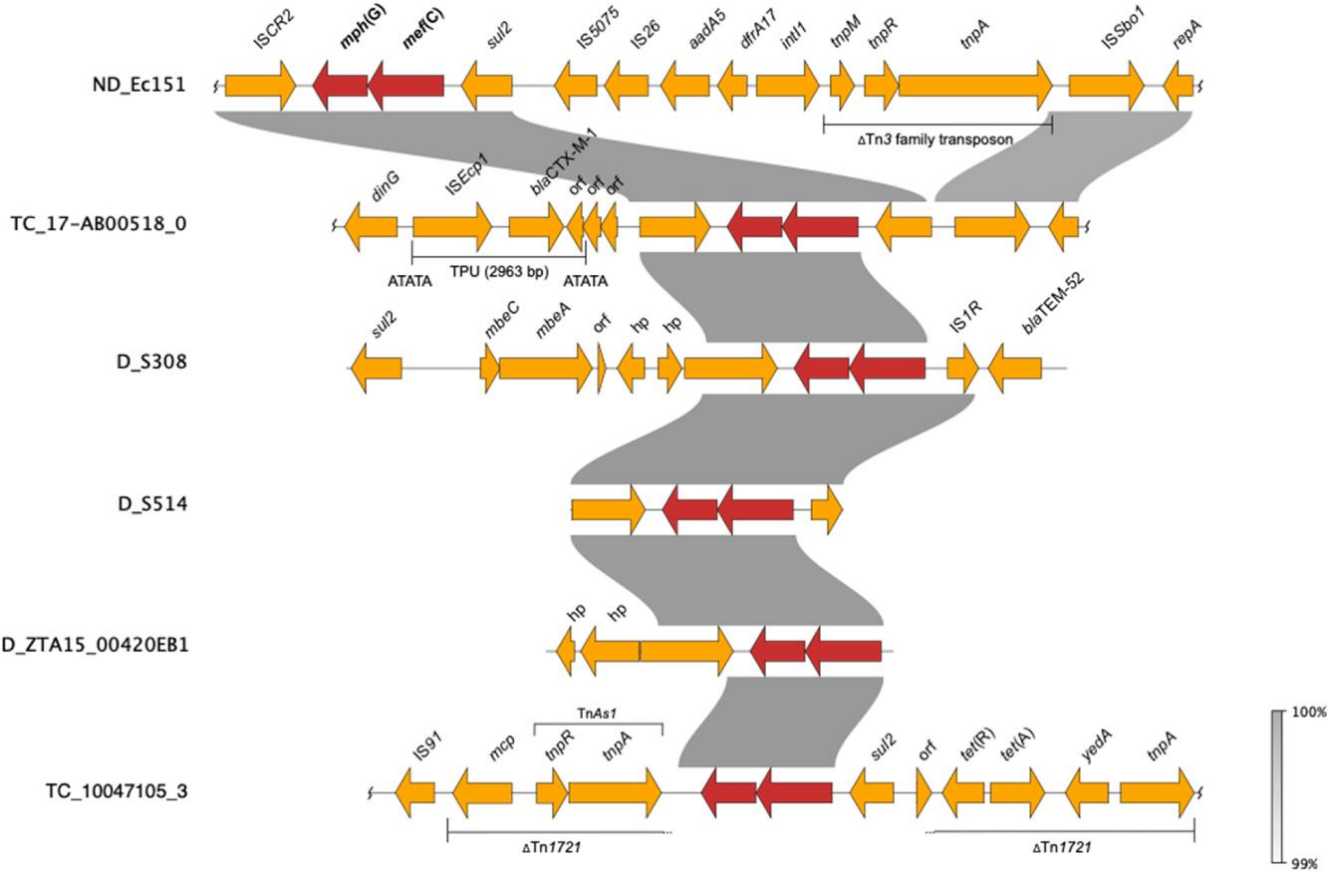
Comparative analyses of contigs harbouring *mef*(C)-*mph*(G) in *E. coli* and *Salmonella*. D, donor (included for isolates that did not yield TCs), ND, conjugation not performed. Wavy lines at the end(s) of diagrams show where only part of the contig is included. This figure appears in colour in the online version of *JAC* and in black and white in the print version of *JAC*.

Exceptions to the *mph*(A) operon structure that is typical in the susceptible isolates [truncated regulatory region and lack of *mphR*(A)] were also observed. The regulatory region was intact in 13 isolates, and in 5 of them *mphR*(A) was absent, whereas in 7 of them *mphR*(A) was present but *mrx* had a deletion mutation at nucleotide position 576 leading to a premature stop codon (PMSC) and truncated Mrx. However, previous cloning experiments in minicells carrying *mrx* with a non-sense mutation showed that Mph(A) production was still enhanced in presence of erythromycin as in minicells carrying non-mutated (WT) *mrx* gene.^29^ Therefore, the deletion mutation in *mrx* in the seven isolates in our study might not be the explanation for the observed susceptible phenotype. Additionally, in isolate NRS_2017_ESBL_19.14 (MIC = 8 mg/L), *mphR*(A) was present and the regulatory region intact, but *mrx* was located in two contigs. In isolate U16-0318X (MIC = 8 mg/L) *mph*(A) had a deletion (Figure 3c, 4).

Despite exceptions to each of the two most common *mph*(A) operon structures, the size of the dataset analysed and the high concordance between *mph*(A) operon structures and observed phenotypes suggest that in addition to the *mph*(A) gene, the *mphR*(A) repressor gene is also required for azithromycin resistance. Nevertheless, *in vitro* studies are needed to elucidate the association between the absence of *mphR*(A), truncation in the regulatory region and susceptibility to azithromycin.

### mef(C)-mph(G) genes

The *mef*(C)-*mph*(G) tandem genes, conferring high-level azithromycin resistance, were first identified in *Photobacterium damselae* subsp. *damselae*^31^ on an MDR plasmid pAQU1 and subsequently found in other marine and enteric bacteria from fish intestines.^31,32^ Since their first report in Enterobacterales, in a Shiga toxin-producing *E. coli* (STEC) isolate in 2021,^9^  *mef*(C)-*mph*(G) have been detected in *E. coli* from various origins and geographical locations.^33–36^

We first detected *mef*(C)-*mph*(G) in *E. coli* and *Salmonella* isolates included in the 2018 EU harmonized monitoring of AMR and the Danish Programme for surveillance of antimicrobial consumption and resistance (results included in this study). Here, screening of all *E. coli* and *Salmonella* isolates collected between 2014 and 2018 identified *mef*(C)-*mph*(G) in 11 *E. coli* and two *Salmonella* isolates from bovine and porcine origin. In all isolates, *mef*(C)-*mph*(G) were associated with an azithromycin-resistant phenotype (MIC > 16 mg/L). No other known macrolide resistance mechanisms were detected in these isolates, except *mph*(B) in isolate ZTA15:00420EB1 (Tables 1 and S4). However, other AMR genes were found in the *mef*(C)-*mph*(G)-harbouring isolates, i.e. *tet*(A), *sul2*, *bla*_TEM-52_ and *bla*_CTX-M-1_, in all cases located on the same contig as *mef*(C)-*mph*(G) (Figure 4). The *E. coli* isolates carrying *mef*(C)-*mph*(G) belonged to eight different STs, and the two *Salmonella* isolates, *S*. Derby and *S*. 1,4, [5],12:i:-, belonged to ST40 and ST19, both of which have frequently been associated with the pork production chain (Table 1).^37,38^

Since *mef*(C)-*mph*(G) genes were previously described to be located on plasmids and associated with various mobile genetic elements, including plasmids of various sizes and integrative conjugative elements,^9,32^ we carried out conjugation experiments to assess their transferability. The conjugation performed for 10 *E. coli* and two *Salmonella* isolates showed that *mef*(C)-*mph*(G) could be located on transferrable plasmids, as in 5 of the *E. coli* isolates the genes were successfully transferred to the *E. coli* J62-2 recipient (Table 1). Unequivocal association between *mef*(C)-*mph*(G) and a plasmid replicon gene could not be made using short-read sequencing data. However, in all TCs, except TC_NRS-2015 ESBL-08-69, only one plasmid replicon was detected, which allowed us to associate *mef*(C)-*mph*(G) with IncFII in isolate TC_10047105-3 and IncIγ in isolates TC_17-AB00518-0, TC_HP-6957 and TC_ECO-NRS-187-57 (Table 1). Analyses of the genetic contexts of *mef*(C)-*mph*(G) in the donor strains and TCs revealed that the genes were associated with truncated Tn*As1* consisting of *tnpR* and *tnpA* genes in IncFII plasmids and IS*CR2* in IncIγ plasmids, with both elements located downstream of *mef*(C)-*mph*(G) (Figure 4). The IS*CR2* is known to be associated with dissemination of multiple resistance genes in various species.^39^

### Other azithromycin resistance genes

The relevance of the plasmid-borne *erm* genes in mediating high-level azithromycin resistance was confirmed in this study as previously described.^7,8,35,40^ The presence of *erm*(C), *erm*(B) and *erm*(42), alone or in combination with other macrolide resistance genes, was associated with a resistant phenotype (MIC ≥ 64 mg/L) in all cases. Association of *erm* genes with high azithromycin MIC levels has been reported in *E. coli* even in the presence of efflux pump inhibitors.^6,41,42^

The *mph*(B) gene, the second most detected gene in our collection, encodes macrolide 2′-phosphotransferase II, which phosphorylates 14-membered (e.g. erythromycin) and 16-membered (e.g. tylosin) macrolides.^43,44^ Nevertheless, 6 out of 26 isolates harbouring *mph*(B) as the sole macrolide resistance gene in our collection had azithromycin MIC > 16 mg/L (Table S4). The only *mph*(B)-harbouring isolate in the study of Gomes *et al*.^7^ exhibited an MIC of 16 mg/L in the absence of the efflux pump inhibitor PaβN. Previous cloning and expression analysis of *mph*(B) in *E. coli* also showed that *mph*(B) did not confer resistance to azithromycin.^44^

In addition, we detected *mef*(B), *msr*(E)-*mph*(E) or *ere*(A) as the only macrolide resistance gene in isolates with azithromycin MIC > 16 mg/L. While azithromycin-resistant *ere*(A)-carrying *E. coli*, even in the presence of PaβN, has been observed,^7^ there is no description of *msr*(E)-*mph*(E) or *mef*(B) conferring azithromycin resistance in *E. coli* or *Salmonella*. Generally, the decreased azithromycin susceptibility in isolates with MIC = 32 mg/L without a known mechanism of azithromycin resistance could be explained by the one 2-fold dilution MIC variation from the cut-off for resistance. However, the occurrences of high-level azithromycin resistance (MIC ≥ 64 mg/L) that cannot be explained by genes detected in this study suggest that unknown mechanism(s) could be involved in the reduced azithromycin susceptibility. For instance, a recent study using hidden Markov models identified and experimentally validated five novel macrolide resistance genes that increased the azithromycin resistance when cloned into *E. coli*.^45^

## Study limitations

A limitation in our study was that we could not unequivocally determine whether the detected macrolide resistance genes were located on the chromosome or on plasmids due to the nature of the Illumina short-read sequencing technology. Furthermore, the association of AMR genes with IS or Tn elements is not entirely certain, due to the possibility of mis-assemblies, although studies suggest that these tend to be rare for high-coverage data. Both limitations could be overcome by long-read sequencing technologies.

### Conclusions

We investigated azithromycin resistance determinants and phenotype–genotype correlation in a collection of 1276 *E. coli* and *Salmonella* isolates from food-producing animals and meat in Europe. Eleven different genes previously associated with azithromycin resistance were detected. Our study highlights that the interpretation of *mph*(A)-mediated phenotypes in Enterobacterales requires consideration of the entire *mph*(A) operon and its regulatory region. Generally, azithromycin-susceptible isolates lacked the *mphR*(A) repressor gene and had a truncated *mphR*(A) binding site, while azithromycin-resistant isolates had an intact *mph*(A) operon and complete *mphR*(A) binding site. We observed elevated azithromycin MIC values (≥64 mg/L) in all cases when an *erm* gene was found alone or associated with another macrolide resistance determinant. Moreover, our study provides additional insight into the genetic context and transferability of *mef*(C)-*mph*(G) genes, which are emerging in *E. coli* and *Salmonella*. These genes were found to be associated with various transposable elements, often harboured on conjugative plasmids and containing additional AMR genes, thus highlighting concerns for co-selection and spread.

## Supplementary Material

dkae161_Supplementary_Data

## Supplementary data

Tables S1–S6 are available as Supplementary data at *JAC* Online.
